# Medical Negligence and Duty of Care in Nepal: A Legal and Ethical Analysis

**DOI:** 10.31729/jnma.9171

**Published:** 2025-07-31

**Authors:** Kaschev Shrestha, Jyotsana Joshi, Dhirendra Yadav

**Affiliations:** 1 Department of Forensic Medicine, College of Medical Sciences, Bharatpur, Chitwan, Nepal; 2 College of Medical Sciences, Bharatpur, Chitwan, Nepal; 3 Patan Academy of Health Sciences, Lagankhel, Lalitpur, Nepal

**Keywords:** *duty of care*, *informed consent*, *institutional accountability*, *medical egligence*, *standard of care*

## Abstract

Medical negligence, a critical intersection of ethical obligations and legal standards, is an evolving concern in Nepal’s medico-legal landscape. This article explores the concept of duty of care, which is fundamental to medical negligence, and emphasizes the need for a robust legal framework to ensure justice for both the patients and the healthcare professionals. The key elements of negligence: duty, dereliction, direct causation, and damage are examined alongside international precedents like Donoghue v. Stevenson, Bolam v. Friern Hospital, Bo-litho v. City and Hackney and Montgomery v. Lanarkshire, which shape standards of care and informed consent. In Nepal, cases such as the Infant Amputation Case, Dr. D.B. Shah v. Srijana KC and the Sterilization Malpractice Case highlight the growing recognition of patient rights and institutional accountability. This article advocates for a dedicated Medical Negli-gence Act to strengthen legal protections, emphasizing expert testimony, informed consent, and institutional responsibility to foster a balanced, ethical, and just healthcare system.

## INTRODUCTION

Medical negligence is a critical issue that combines ethical obligations and legal standards, hence requiring a strong comprehension of the duty of care. In the Nepalese context, where the subject of medical jurisprudence is evolving, there is a need to examine the laws applicable in the event of medical negligence in order to deliver justice to both the medical professionals and the patients.^1^ Medical negligence happens when a healthcare practitioner fails to meet the standard of care and therefore causes injury to the patient.

## UNDERSTANDING DUTY AND STANDARD OF CARE

The principle underlying the law of medical negligence is the duty concept, or the responsibility of medical professionals to take reasonable care in the delivery ofmedical care. The Blyth vs. Birmingham Waterworks Co. case established the fundamental elements of the negligence law, including the requirement to follow accepted standards of medicine so as to avert injury.^2^

Similarly, Slater v. Baker and Stapleton highlighted the importance of informed consent and standardized care in medical practice.^3^

In the law of negligence, a successful claim for medical negligence necessitates the establishment of four fundamental elements: duty, dereliction of duty, direct causation and damage.

1. Duty—The obligation to exercise reasonable care.
2. Dereliction of Duty—A breach in the standard of care.
3. Direct Causation—The relationship between negligence and subsequent injury.
4. Damage—The real loss or injury incurred due to negligence.

## DETERMINING THE STANDARD OF CARE

The standard of care is the test of medical practice, and this test determines if professional duty has been satisfied. Here, are some of the international cases that are pivotal for the evolution of determining standard of care.

**1. Donoghue v. Stevenson (1932)** This case started when Mrs. Donoghue became ill after finding a dead snail in a bottle of ginger beer which she had been drinking. She sued the manufacturer, claiming that even though she didn’t buy directly from them, they still had an obligation to make sure it was safe. Hence, this case formulated the principle of reasonable care, extending it to professional duties, including medical practice.^4^

**2. Bolam v. Friern Hospital Management Committee (1957)**

The reason for this case was that Mr Bolam, a patient receiving electroconvulsive therapy (ECT), sustained severe fracture. He filed lawsuit against the hospital, claiming that it was negligent for failing to provide muscle relaxants, restrain him and inform him of procedures risk. This case established the Bolam test, which states that a medical practitioner is not negligent if his actions are in accordance with the standards of a responsible body of medical men. Nevertheless, critics argue that this evaluation relies too heavily on peer reviews at times, at the expense of patient safety^5^

**3. Bolitho v. City and Hackney Health Authority (1997)**

The cause of this case was the death of two years old child who had croup. A doctor was called but she didn’t show up because her pagers battery was low. The mother of the child filed the lawsuit, claiming that the child’s life would have been saved if the doctor had come and intubated her child. To address this limitation concerning medical negligence, this case established the necessity for medical opinions to be logically defensible rather than merely accepted by the majority.^6^

**4. Montgomery v. Lanarkshire Health Board (2015)** The case was started by Ms. Montgomery, a diabetic woman who had given birth to a severely disabled son as a consequence of shoulder dystocia; and she alleged that the doctor was negligent for failing to inform her about the higher risk of shoulder dystocia in diabetic mother, which would have given her the choice to opt for a caesarean section. The case put much emphasis on patient autonomy and informed consent. The case reiterated that doctors have to provide patients with all the information that is relevant to risks, rather than unilaterally deciding what the patient needs to be informed about.^7^.

## MEDICAL NEGLIGENCE IN NEPAL: LEGAL PRECEDENTS AND CHALLENGES

Nepal’s jurisprudence has increasingly acknowledged the importance of medical negligence, although its jurisprudence is still evolving. The following leading cases have influenced medical negligence law in Nepal:

**1. Case No. 072-CI-0548, 0552, 0549 (Infant Amputation Case)** ^8^

Here, the District Court awarded damages regarding the suspected negligent amputation of a limb from a preterm infant. In the appellate case, the higher court noted the importance of an expert’s opinion, encapsulating the changing attitude of Nepal towards medical malpractice claims.

**2. Dr. D.B. Shah v. Srijana KC (Pro-Public) 2066** ^9^

The Supreme Court reaffirmed the informed consent doctrine, ruling that medical professionals have a duty of full disclosure regarding treatment alternatives, attendant risks and expected results prior to seeking the consent of patients. This is in line with global best practice and therefore strengthens the right of patients to make an informed decision on matters pertaining to their health.

**3. Supreme Court Case DN 10061 (Sterilization Malpractice Case)** ^10^

In this case, a hospital was held liable for its failure to ensure proper sterilization procedures, which in turn led to septicaemia in a patient. Despite the due care exercised by the medical staff, the institution was held responsible for its failure to exercise proper infection control measures. This case set an important precedent with respect to institutional liability in cases of medical negligence.

## THE WAY FORWARD: FORTIFYING LEGAL AND ETHICAL PROTECTIONS

Considering the increasing cases of medical negligence, the Supreme Court of Nepal has directed the government to introduce a special Medical Negligence Act to provide a clearer legal framework and protect both patients and practitioners. The directive states that:

- The necessity for professional testimonies in cases of medical negligence is critical to maintain fairness in judicial proceedings.
- Fortifying the doctrine of informed consent. - Requiring physicians to record patient consent and give detailed risk disclosures.
- Institutional responsibility for healthcare service quality- holding healthcare institutions and hospitals, rather than individual medical practitioner employed there, responsible for systemic failure at the healthcare providing centres.

Though ignorance of legal statutes is not an excuse, the evolving nature of medical science and patient rights calls for continued discussion on legal and ethical issues. By tempering legal accountability with the realities of medicine, Nepal can craft a fair and functional medico-legal system that safeguards patient rights without unjustly subjecting good-faith healthcare providers to unwarranted legal ramifications.

## CONCLUSION

Negligence goes beyond law—it’s a humanitarian issue because it impacts trust in health care systems. While Nepal is at a critical stage of developing its medico-legal environment, there is a need for striking the right balance between patient protection and the protection of well-intentioned medical practitioners. The dedicated Medical Negligence Act, along with the adherence to legal principles of other countries with advanced and trusted health care systems, would contribute significantly towards the establishment of a just, responsible and effective system of medical practice in Nepal. This will provide legal responsibility, ethical respect and institutional accountability, which will then enable Nepal to establish a safe and professional environment in the health care system.

## References

[1] Adejumo OA, Adejumo OA (2020). Legal Perspectives on Liability for Medical Negligence and Malpractices in Nigeria.. Pan Afr Med J..

[2] Blyth v Birmingham Waterworks Co. [Internet]. (2025). Casebriefs.

[3] Miller RD (2019). Slater v Baker and Stapleton (C. B. 1767): Unpublished Monographs. [Internet]..

[4] Blyth v Birmingham Waterworks Co. (2024). In: Wikipedia [Internet]..

[5] Chaiyasate S, Salee P, Sukapan K, Teeranoraseth T, Roongrotwattanasiri K (2020). Rhinofacial Entomophthoramycosis Case Series, The Unusual Cause Of Facial Swelling.. Annals Of Medicine And Surgery.

[6] Edersheim JG, Stern TA (2009). Liability Associated With Prescribing Medications.. Prim. Care Companion J. Clin. Psychiatry..

[7] Skoczek A, Prochownik P, Gancarczyk U, Libiszewska N, Podolec P, Komar M (2019). Personality Traits Of Patients Suffering From Congenital Heart Defects.. Wiad Lek..

[8] Infant Amputation Case, Case No. 072-CI-0548, 0552, 0549. https://supremecourt.gov.np/special/syspublic.php?d=reports&f=case_details.

[9] Dr D.B. Shah v Srijana KC (Pro-Public), Case No. 20669. https://supremecourt.gov.np/lic/sys.php?d=reports&f=casedetails.

[10] Sterilization Malpractice Case, Case No. DN 10061. https://supremecourt.gov.np.

